# Effect of public hospital managers’ risk and gain perception on their attitude towards physician dual practice: a cross-national study in 31 provinces of China

**DOI:** 10.1186/s12889-020-09207-1

**Published:** 2020-07-13

**Authors:** Yuanyuan Yu, William C. S. Cho, Qiaoman Liu, Xiaojing Fan, Xiaolin Chen, Xihe Yu, Yijun Lv, Xiumin Zhang

**Affiliations:** 1 School of public health, Jilin University, No.1163 Xinmin Street, Chaoyang District, Changchun, 130000 Jilin Province China; 2 First Affiliated Hospital of Wenzhou Medical University, Nanbaixiang, Ouhai District, Wenzhou, 325000 Zhejiang Province China; 3 Department of Clinical Oncology, Queen Elizabeth Hospital, Hong Kong, SAR China; 4 Jiangsu Vocational Institute of Commerce, No.180 Longmian Street, Jiangning District, Nanjing, 210012 China; 5 School of Public Policy and Administration, Xi’an Jiaotong University, No. 28 Xianning West Road, Xi’an, 710049 Shaanxi China; 6 Wenzhou Medical University, Nanbaixiang, Ouhai District, Wenzhou, 325000 Zhejiang Province China

**Keywords:** Physician dual practice, Determinants, Public hospital, China

## Abstract

**Background:**

This study aims to explore the effect of public hospital managers’ risk and gain perception on their attitude towards physician dual practice (PDP).

**Methods:**

A cross-sectional study enrolling 1513 managers from public hospitals in the East, Middle and West of China was conducted. Generalized linear mixed models (GLMM) were used to identify the determinants of managers’ support for PDP.

**Results:**

The rate of managers’ support for allowing PDP or implementing PDP with restriction, was 94.3% (95% CI: 0.93, 0.95). The mean score of managers’ risk perception was 67.7 ± 14.46, and the mean score of managers’ gain perception was 24.0 ± 5.56. After controlling for individual and institutional characteristics, the GLMM presented the score for risk perception increased 1 score and the rate of managers’ support for PDP decreased by 5% (OR = 0.95, 95% CI: 0.93, 0.97); while the score for gain perception increased 1 score and the rate of managers’ support increased by 18% (OR = 1.18, 95% CI: 1.12, 1.24).

**Conclusions:**

Our data demonstrate that the majority of Chinese public hospital managers are in favor of allowing or implementing PDP with restrictions. Although gain perception is comparatively weaker than risk perception, a stronger influence in determining public hospital managers’ support for PDP is demonstrated.

## Background

In contrast to population health care needs, health care service provision is in shortage, which is quite common in both developed and developing countries [1, 2]. Hence, physician dual practice (PDP), a phenomenon that a physician can perform clinical practice in both the public and private sectors [3–5], is believed to provide greater access to health care services for more patients [6–8]. According to common opinions from 15 of the world’s top medical experts and health policy makers, as well as from evaluations from the World Health Organization (WHO), PDP ranks the second among global health care human resources research concerns [9]. Through PDP, physicians can increase their income and professional satisfaction [10]. PDP may also reduce the financial burden on governments to retain high quality medical service in the public sector [7, 11]. However, debates on the disadvantages of PDP never cease, and negative effects of physicians engaging in public sectors and private sectors by far exceed the positive [12, 13]. Considering limits of the resources, physicians engaging in PDP may shirk their public sector duties due to competition for time, and reduce the quantity or quality of their services in public sector due to conflict of interest [14]. Furthermore, PDP also gives opportunity to hidden outflow of public sector resources, especially highly skilled physicians, rich and low complicated patients [15]. Therefore, further exploration and investigation are needed as effect might change in different contexts [5].

**Fig. 1:**
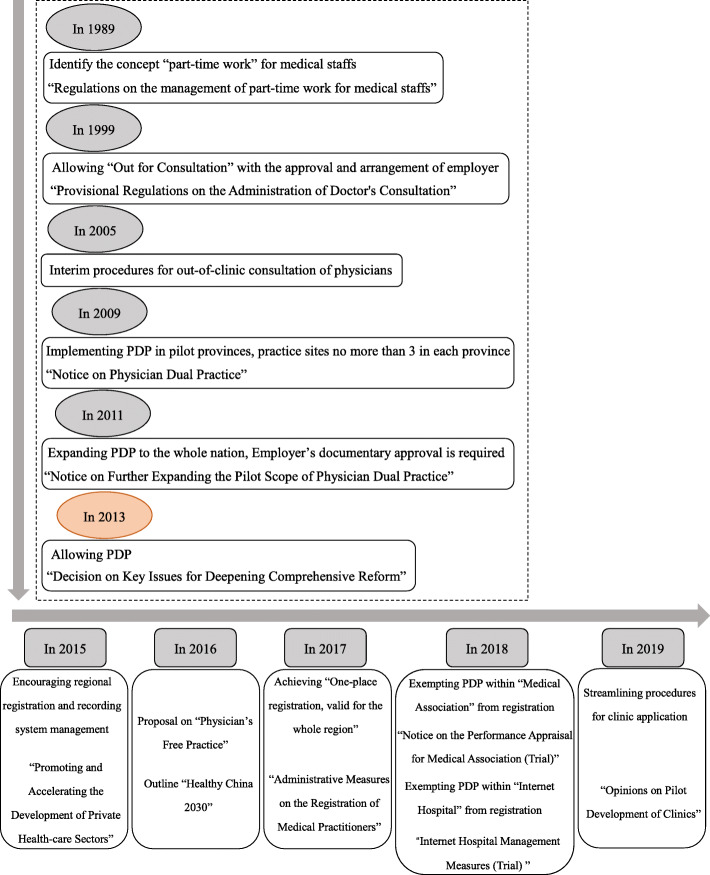
The flow chart of physicians’ dual practice policies

In 2009, China initiated new health-care reform with aims of achieving accessible and affordable health care service with full coverage for the entire population [16]. As a result, “Public Hospital Reform” and “Encouraging Private Hospital” became more important than before. Acting as a bridge between the public and private sectors, PDP draws great attention from both the government and general public for the equity, efficiency and quality of health care provision [17]. The policies and regulations on PDP underwent favorable changes in the last decade (Fig. 1). However, PDP did not enter a booming stage as expected, and substantial there are hot debates on how PDP impacts public hospitals’ performance has ensued [10]. According to a survey of 35,500 Chinese physicians, 50% of practitioners did not participate in the PDP due to the reluctant attitudes public hospitals towards PDP [18]. This finding is supported by a previous study, which identified that 71.6% of the Chinese physicians reported that the resistance to PDP originated from the administration of the public hospitals [19]. In China, public hospitals take most of the responsibilities for their physicians professional development, and invest a lot during physicians career life. In turn, physicians make public hospitals trustworthy and the priority for patients. However, the intrinsic characteristic of PDP is mobility, which threatens the interdependence between public hospitals and their physicians. Moreover, PDP greatly challenges the continuity and constancy of public hospitals’ personnel management, performance appraisal, compensation system, social security payment, medical risk insurance etc., thus affects the supervision capacities of public hospitals and increases their administration costs [20]. Hence, it is important to investigate the public hospital managers’ attitude towards PDP, as well as the determinants.

This study which analyzed survey data collected in the East, Middle and West of China, aims to investigate the determinants influencing public hospital managers’ support for PDP by using risk and gain perspectives that are based on valence-instrumentality-expectancy (VIE) theory. VIE theory is widely applied in psychological and behavioral studies to explain decision process and job performance, with a three-way interaction between (a) the importance attached to certain outcomes (Valence), (b) the extent to which performance is believed to result in these outcomes (Instrumentality), and (c) the extent to which extended effort is believed to result in effective performance (Expectancy) [21, 22]. In other words, an individual’s decision is given by the sum of positive and negative values (Valences), individual attaches to outcomes and individual performance-outcome probability (Instrumentality) multiplied by individual effort-performance probability (Expectancy). VIE theory argues that role perceptions of positive and negative values are important determinants of job performance. This study hypothesizes that a managers’ risk perception (a negative value), as well as their gain perception (positive values), could influence their decision in supporting PDP in a public hospital setting.

## Methods

### Data source

From January to October in 2019, we conducted a hospital-based survey with coverage of 31 provinces in the East, Middle and West of China. Managers of public hospitals who signed the informed consent were enrolled. This study used a convenience sample, at a series of meetings, conferences, and training sessions organized by the National Health Commission for public hospital managers, including: China Modern Hospital Management Seminars, National Tertiary Public Hospital Performance Evaluation Training Courses, Conference of Experience Exchange on China’s Medical Reform, Conference of Experience Exchange on Hospital Discipline Development in China and so on. Presidents, vice-presidents in charge of clinical and medical affairs, and directors of medical affairs departments in China public hospitals were included in this study. In total, 1513 questionnaires were completed.

### Development of the instrument

The risk and gain perception of managers was assessed based on a questionnaire (Supplementary material 1). The questionnaire developed as follows: 1) the two variables of risk and gain perception, derived from consumer purchase decision research, were modified according to the specific scenarios of PDP; 2) two rounds of expert consultation were conducted to make preliminary amendments to the initial scale. The expert group was composed of the managers from the Medical Administration Bureau and public hospitals, professors in the public health sectors, and doctors with a professional title. Items on the scale of risk and gain perception were discussed and measured carefully by experts before the questionnaire was finally approved; 3) a pre-survey was conducted with a sample of 252 managers from public hospitals in Wenzhou city and Hangzhou city; 4) homogeneity test and the change of Cronbach’s α coefficient were both applied for item selection. The homogeneity test was used to calculate the correlation value (Corrected Item-Total Correlation, CITC) between each item’s score and the total score of the scale as a criterion for deleting the item [23]. The CITC value of each item was higher than 0.5, indicating that all the items of manager’s risk and gain perception variables were highly homogeneous. Finally, Cronbach’s α was used as a test for reliability [24], and confirmatory factor analysis was used as a validity test of the questionnaire [25].

### Study variables and sample size

Managers were asked to evaluate their risk perception by answering 20 questions (Section B in Supplementary material 1) and their gain perception by answering 8 questions (Section C in Supplementary material 1) using a 5-step Likert scale. The answers were reported as: completely disagree (score 1), less agree (score 2), neutral (score 3), more agree (score 4) or completely agree (score 5). The outcome variable was managers’ support for PDP (the first question in Section E). This category variable was divided between completely prohibited, and permitted (including allowing with restrictions and completely allowing). In this study, binary variables (0 = completely prohibited; 1 = permitted) were used for the convenience of explaining a managers’ support for PDP. According to the results of a previous study, the rate of support for managers for PDP was 44.5% [26]. Therefore, the sample size was calculated as 673 with a permissible error of δ = 3%, a type I error of α = 0.05 and an expected 20% non-response rate. We recruited a total of 1513 managers from the East, Middle and West of China in the public hospitals which met the requirement for statistical analysis.

### Quality control

In order to ensure the results of the survey were accurate and reliable, we passed the results to experts with statistics expertise and experience of field research. Prior to the formal survey, research assistants were trained in accordance with a unified training plan and teaching materials. The training content included: the main purpose of the research, content, related terminology of this study and duration of this investigation; instructions for questionnaire filling and guidance for each item; explanations that may be necessary at the scene. This protocol ensured that all research assistants were familiar with the purpose and techniques required for survey, the meaning of the indicators and the content of the questionnaire before data collection. Each research assistant underwent a rigorous simulated training session before formal data collection. Regular monitoring of data collection was conducted throughout the data collection process.

### Statistical analysis

In this study, all records have been checked for any missing data or outliers. The missing data in managers’ support for PDP (outcome variable) and outliers in all selected variables were excluded prior to the data analysis. The scores of risk perception and gain perception were described with means and standard deviations of categorical variables including: sex, age, education, major, length of service, level of managers’ position, institutional category, institutional level, institutional territory and location, which were ultimately summarized as percentages. The rates of managers’ support for PDP were compared with Chi-squared test to identify the determinants. Generalized linear mixed models (GLMM) including fixed and random effects were used in this study to identify the determinants of managers’ support for PDP when controlling for other confounding factors. The scores of risk and gain perception were specified as fixed effects and managers’ work place as a random effect; managers’ sex, age, education, major, length of service, level of managers’ position, institutional category, institutional level, institutional territory and location were included as covariates (the detailed description was showed in Table 1). There are three models to explore the determinants: Three GLMMs were established from odds ratios (OR) together with 95% confidence intervals (CIs) for each determinant regarding managers’ support. Model 1 included the demographic determinants of managers’ sex, age, and education. Model 2 included demographic determinants from model 1 plus managers’ educational major, length of service, and level of managers’ position. Model 3 included determinants from models 1 and 2 in addition to work environment determinants of managers’ institutional category, institutional level, and institutional territory and location. All analyses were performed with STATA statistical software version 12.0 (StataCorp LP, College station 77,845, USA). A two-tailed *P* value < 0.05 was considered statistically significant.

**Table 1:** List of variables and description

Variables	Description
**Demographic characteristics**	
Sex	0: male; 1:female
Age (years)	1: aged less than 40 years old; 2: aged between 41 years old and 50 years old; 3: more than 51 years old
Education	1: PhD; 2: Master; 3: Bachelor; 4: Others
**Work characteristics**	
Major	0: other majors (including administration, technology, economics, management, et al); 1:medicine
Length of service (years)	1: works less than 5 years; 2: works between 6 years and 10 years; 3: more than 11 years
Level of manager’s position	0: manager’s position is less than deputy division; 1: manager’s position is more than county level (including province level, city level, county level)
**Working environment characteristics**	
Institutional category	0: means special hospital, Chinese medicine hospital and others; 1:means general hospital
Institutional level	0: means primary level and second level; 1:means tertiary level
Institutional territory	1: east of China; 2:middle of China; 3: west of China
Location	0: means manager coming from provincial capital city; 1:means manager coming from non-provincial capital city

## Results

### Reliability and validity of questionnaire

In this study, the Cronbach α coefficient of managers’ risk and gain perception was more than 0.7, which met the internal consistency requirements of the instruments. As a confirmatory factor analysis demonstrated, from the perspective of the standardized factor load of each measurement item corresponding to the risk and gain perception, both of confirmatory factor analysis were higher than 0.5, with *P* value less than 0.001; CR was greater than 0.72; and AVE was greater than 0.46. Therefore, the risk and gain perception could achieve effective convergence aggregation, and the scale had good convergence validity.

### Demographics of managers in the public hospital

The demographics of the sample of managers are listed in Table 2. There were 893 male managers and 623 female managers. About 57.44% of them majored in clinical medicine, including surgery, internal medicine, paediatrics, gynaecology, ophthalmology, otorhinolaryngology, dermatology, orthopaedics, psychiatry, traditional chinese medicine and general practice. Approximately 77% of the sample were under 50 years old and 51.51% had worked in the current positions for more than 6 years. Most of the managers’ positions were lower than county level (77.86%) with education levels higher than a baccalaureate (95.77%). 37.91% of the sample were from the East of China, 29.69% from the Middle of China, and 32.40% from the West of China. 94.74% of the sample worked in a public tertiary hospital, the primary source of PDP.

**Table 2:** Basic characteristics of managers (*n* = 1513)

Variables	n	%
**Demographic characteristics**		
Sex		
Male	893	59.02
Female	620	40.98
Age (years)		
≤ 40	452	29.87
41–50	713	47.13
≥ 51	348	23.00
Education		
PhD	72	4.76
Master	398	26.31
Bachelor	979	64.71
Others	64	4.23
**Work characteristics**		
Major		
Others	644	42.56
Medicine	869	57.44
Length of service (years)		
≤ 5	628	48.49
6–10	338	26.10
≥ 11	329	25.41
Level of manager’s position		
≤ Deputy division	1178	77.86
≥ Country level	335	22.14
**Working environment characteristics**		
Institutional category		
Others	537	34.49
General hospital	976	64.51
Institutional level		
< tertiary level	72	5.26
tertiary level	1298	94.74
Institutional territory		
East	572	37.91
Middle	448	29.69
West	489	32.41
Location		
Provincial capital city	525	34.70
Non-provincial capital city	988	65.30

### Distribution of different perception of managers’ support for PDP

The mean score of managers’ risk perception was 67.7 ± 14.46 and reflected differences in terms of managers’ sex, age, major, length of service, institutions category, institutional level and location of managers’ hospital (*P* < 0.05). The mean score of risk perception for female managers was 68.8 ± 13.49, which was higher than for male managers (66.9 ± 15.05). With increasing age, the mean score of risk perception increased, with managers aged 51 years old having the highest score for risk perception (68.7 ± 15.30). Managers from majors other than clinical medicine (69.6 ± 13.46) and those with longer lengths of service (6–10 years: 67.4 ± 15.18; ≥ 11 years: 67.8 ± 15.50) held higher risk perception.

In contrast, the mean score of managers’ gain perception of 24.0 ± 5.56 was correlated with managers’ age, length of service and institutional level (*P* < 0.05). The score of managers’ gain perception was highest in managers aged less than 40 years old (24.5 ± 4.96). Managers with length of service of 6–10 years held higher gain perception scores than those with less than 5 years or more than 11 years of service. It is noteworthy that managers in tertiary hospitals had a higher risk perception (68.6 ± 14.24, *P* < 0.001), but lower gain perception (23.8 ± 5.51, *P* = 0.003) than managers from public hospitals. (Table 3).

**Table 3:** Descriptive of total score of different perception of managers’ support for physician dual practice

Variables	Risk perception			Gain perception		
	Mean	Std.Dev	*P*	Mean	Std.Dev	*P*
**Demographic characteristics**						
Sex			0.006			0.240
Male	66.9	15.05		24.1	5.66	
Female	68.8	13.49		23.9	5.42	
Age (years)			0.004			< 0.001
≤ 40	33.8	13.13		24.5	4.96	
41–50	67.8	14.83		23.9	5.68	
≥ 51	68.7	15.30		23.6	6.03	
Education			0.087			0.315
PhD	66.2	15.40		24.7	5.75	
Master	66.7	13.40		24.0	5.27	
Bachelor	68.0	14.80		24.0	5.68	
Others	71.3	13.50		23.5	5.30	
**Work characteristics**						
Major			< 0.001			0.058
Others	69.6	13.46		23.7	5.32	
Medicine	66.3	15.01		24.2	5.73	
Length of service (years)			0.032			0.029
≤ 5	67.3	13.85		23.9	5.34	
6–10	67.4	15.18		24.3	5.57	
≥ 11	67.8	15.50		23.7	6.06	
Level of manager’s position			0.433			0.362
≤ Deputy division	67.7	14.36		24.0	5.42	
≥ County level	67.8	14.80		23.9	6.05	
**Working environment characteristics**						
Institutional category			0.007			0.157
Others	68.9	13.53		23.8	5.63	
General hospital	67.0	14.90		24.1	5.52	
Institutional level			< 0.001			0.003
< tertiary level	59.4	15.66		25.7	6.39	
tertiary level	68.6	14.24		23.8	5.51	
Institutional territory			0.322			0.896
East	66.3	14.10		24.0	5.55	
Middle	70.0	14.10		23.5	5.48	
West	67.3	14.9		24.4	5.60	
Location			0.004			0.076
Provincial capital city	66.4	14.78		24.3	5.69	
Non-provincial capital city	68.4	14.24		23.8	5.49	

### Distribution of demographics of managers among the rate of support for PDP

The rate of managers’ support for allowing PDP and implementing PDP with restrictions was 94.3% (95% CI: 0.93, 0.95). As listed in Table 4, the rate of managers’ support for PDP demonstrated differences among age and institutional territory (*P* < 0.05), while managers’ sex, education, major, length of service, level of managers’ position, institutional category, level and location showed no significant difference on the rate of managers’ support for PDP (*P* > 0.05). Managers aged 41–50 years old had the highest rate of support for PDP; 46.60% (*χ*^*2*^ = 11.27, *P* = 0.004). Managers in the East of China had the highest support for PDP(38.39%), compared to those from the Middle of China (28.98%) and the West of China (32.63%) (*χ*^*2*^ = 6.26, *P* = 0.044).

**Table 4:** Distribution of basic characteristic of managers among the rate of support for physician dual practice

Variables	No		Yes		Total	*χ*^*2*^	*P*
	n	%	n	%			
**Demographic characteristics**							
Sex						0.54	0.464
Male	54	62.79	839	58.79	893		
Female	32	37.21	588	41.21	620		
Age (years)						11.27	0.004
≤ 40	12	13.95	440	30.83	452		
41–50	48	55.81	665	46.60	713		
≥ 51	26	30.23	322	22.56	348		
Education						1.26	0.738
PhD	4	4.65	68	4.77	72		
Master	19	22.09	379	26.56	398		
Bachelor	58	67.44	921	64.51	979		
Others	5	5.81	59	4.13	64		
**Work characteristics**							
Major						1.58	0.208
Others	31	36.05	613	42.96	644		
Medicine	55	63.95	814	57.04	869		
Length of service (years)						1.42	0.492
≤ 5	31	44.29	597	48.73	628		
6–10	17	24.29	321	26.20	338		
≥ 11	22	31.43	307	25.06	329		
Level of manager’s position						0.626	0.429
≤ Deputy division	64	74.42	1114	78.07	1178		
≥ Country level	22	25.58	313	21.93	335		
**Working environment characteristics**							
Institutional category						0.669	0.414
Others	27	31.40	510	35.74	537		
General hospital	59	68.60	917	64.26	976		
Institutional level						0.02	0.895
< tertiary level	4	4.94	68	5.28	72		
tertiary level	77	95.06	1221	94.72	1298		
Institutional territory						6.26	0.044
East	25	29.76	547	38.39	572		
Middle	35	41.67	413	28.98	448		
West	24	28.57	465	32.63	489		
Location						0.18	0.668
Provincial capital city	28	32.56	497	34.83	525		
Non-provincial capital city	58	67.44	930	65.17	988		

### Determinants of managers’ support for PDP

Table 5 shows the multivariate analysis of the determinants of managers’ support for PDP by a generalized linear mixed model. According to the model 1, the total score of risk perception, gain perception, and age had a significant effect on the rate of managers’ support for PDP. By controlling demographic characteristics from model 1, when the risk perception increased 1 score, the rate of managers’ support for PDP decreased 6% (OR = 0.94, 95% CI: 0.92, 0.96). By controlling work characteristics and working environment characteristics, the results in model 2 and model 3 remained statistically significant (*P* < 0.001). Based on the results from model 1, when the gain perception increased 1 score, the rate of managers’ support for PDP increased by 17% (OR = 1.17, 95% CI: 1.12, 1.23). The results in model 3 remained statistically significant (*P* < 0.001) after controlling for work and working environment characteristics. Therefore, we can identify that risk perception is negatively correlated with managers’ support for PDP, but gain perception is positively correlated with managers’ support for PDP. The age of managers was significantly correlated with their support for PDP in model 1. The rate of managers’ support decreased 52% for those aged 41–50 years old (OR = 0.48, 95% CI: 0.24, 0.96). By controlling demographic characteristics and work characteristics in model 2, the rate of managers’ support decrease to 59% for those aged 41–50 years old (OR = 0.41, 95% CI: 0.17, 0.97). However, when controlling for the demographic characteristics of work and working environment characteristics, there was no significant association between managers’ age and their support for PDP.

**Table 5:** Multivariate analysis of determinants of managers’ support for physician dual practice by generalised linear mixed model (*n =* 1513)

Variables	Model 1				Model 2				Model 3			
	OR	95% CI		***P***	OR	95% CI		***P***	OR	95% CI		***P***
		Lower	Upper			Lower	Upper			Lower	Upper	
Risk perception	0.94	0.92	0.96	< 0.001	0.94	0.92	0.96	< 0.001	0.95	0.93	0.97	< 0.001
Gain perception	1.17	1.12	1.23	< 0.001	1.18	1.12	1.23	< 0.001	1.18	1.12	1.24	< 0.001
**Demographic characteristics**												
Sex												
Male	1.00				1.00				1.00			
Female	1.40	0.85	2.32	0.187	1.59	0.88	2.86	0.122	1.69	0.91	3.15	0.098
Age (years)												
≤ 40	1.00				1.00				1.00			
41–50	0.48	0.24	0.96	0.038	0.41	0.17	0.97	0.042	0.44	0.18	1.09	0.076
≥ 51	0.47	0.22	1.01	0.052	0.42	0.16	1.11	0.081	0.42	0.15	1.17	0.098
Education												
PhD	1.00				1.00				1.00			
Master	0.83	0.24	2.86	0.769	1.26	0.34	4.64	0.726	1.70	0.44	6.56	0.444
Bachelor	0.83	0.26	2.65	0.757	1.17	0.35	3.95	0.801	1.33	0.37	4.78	0.659
Others	0.78	0.17	3.53	0.748	1.39	0.23	8.33	0.721	1.65	0.25	10.83	0.602
**Work characteristics**												
Major												
Others					1.00				1.00			
Medicine					0.68	0.38	1.22	0.196	0.66	0.35	1.22	0.186
Length of service (years)												
≤ 5					1.00				1.00			
6–10					1.17	0.57	2.42	0.662	1.54	0.71	3.37	0.276
≥ 11					0.89	0.46	1.74	0.743	1.10	0.52	2.35	0.799
Level of manager’s position												
≤ Deputy division					1.00				1.00			
≥ County level					1.72	0.88	3.39	0.115	1.58	0.78	3.21	0.207
**Working environment characteristics**												
Institutional category												
Others									1.00			
General hospital									0.87	0.47	1.59	0.647
Institutional level												
< tertiary level									1.00			
tertiary level									1.32	0.31	5.67	0.705
Institutional territory												
East									1.00			
Middle									0.65	0.26	1.63	0.357
West									0.69	0.26	1.79	0.440
Location												
Provincial capital city									1.00			
Non-provincial capital city									1.12	0.57	2.20	0.733

Model 1 included the demographical determinants of managers’ sex, age, education. Model 2 included determinants in model 1 plus managers’ major, length of service, level of managers’ position. Model 3 included determinants in model 1 and model 2 plus work environment determinants including managers’ institutional category, institutional level, institutional territory and location

## Discussion

In this study, we found that the rate of managers’ support for PDP in China is 94.3%, including support with restrictions as well as complete support, which is consistent with the goals of policy introduced after 2009 [27]. According to the previous report, PDP had direct implications for access, equity, price and quality of the whole nation’s health care service, and thus benefits the whole coverage of universal health [28]. This outcome could possibly explain why most managers in the public hospitals support PDP, as they have realized the advantages of PDP for improving health care service access to those under-served areas in China.

Judging by the results of our study, we are not surprise to find that the managers in the East of China (38.39%) held the highest rate for favoring PDP, followed by those from the West of China (32.63%). While managers from the Middle of China showed the lowest rate for favoring PDP (28.98%). These results may be explained by the extensive and distinguished Electronic Business (E-Business) and Information Technology (IT) industry development in the East, which connects all of China and brings easier access of PDP for both physicians and patients. According to China Internet Hospital Development Research Report 2020, the domestic health care market based on E-Business and IT has experienced an exponential growth. The scale of internet hospitals exceeded 49 billion RMB in 2018, 67.95 billion RMB in 2019, and was expected to exceed 100 billion RMB in 2020, with average daily visits of internet hospitals 2000–3000 [29].

According to our findings, risk perception and gain perception were significantly correlated with managers’ support for PDP. That is, managers’ support for PDP would increase when gain perception was being enhanced or risk perception was being controlled. Risk perception has been well identified as an intuitive and emotional structure closely related to human perception and behavior [30–32]. Obviously, it is important to reduce risk perception of managers. According to white book on medical practitioners in China 2018, involving 44,600 hospitals and 146,200 physicians, the average weekly working hours in tertiary hospitals and secondary hospitals are 51.05 h and 51.13 h respectively, merely 19.2% physicians perceive themselves in good status during self-assessment, and psychological exhaustion of physicians is significantly greater than that of workers from other industries [33]. Constrained by limited time and energy, PDP may present as absenteeism, tardiness, inefficiency, and lack of motivation among public hospitals. So managers worry about physicians engaged in dual practice compromising service delivery. For Chinese policy-makers, promoting PDP through internet may greatly extend the outreach of PDP for physicians on one hand, and strength the capacities of monitoring and supervision for managers on the other. We could expect wide application of IT in health care industry, such as 5G, internet+, artificial intelligence and telemedicine, could pave the way for the popularity of PDP [34], and reduce the risk of physicians skimping of time and efforts in the public hospitals.

More importantly, this study also found that gain perception held more effects on the managers’ support for PDP than risk perception, which may have some implications for the health-care authority in China. Previously, policy makers in China had long been neglecting the public hospital managers’ gain perception towards PDP [35]. It could be largely explained by the negative attitudes of public hospital managers [36]. Many countries have prioritised gain perception [14]. For example, in the U.K., dual practitioners within the National Health Service (NHS) are required to pay 9% of their incomes as compensation to NHS. While in Germany, dual practitioners pay according to what is prescribed in their contracts with public hospitals. On the other hand, many countries also developed effective measures to reduce risk perceptions of public hospitals, such as in France and Ireland, where private practice is encouraged within public hospitals during off-duty time for easier and better monitoring. Again in U.K., physicians of the NHS are required to work at least 4 days per week within public hospitals. While in Australia, public hospitals employ lawyers to lower the negative effects on normal running of the hospital once medical disputes concerning PDP occurred [14]. Therefore, it is effective ways for Chinese policy-makers to establish reasonable benefit distribution mechanism, cost sharing mechanism, economic loss identification and compensation mechanism among the public and private sectors, so as to achieve the public hospital managers’ sense of gain perception.

### Limitations

Firstly, the determinants of managers’ support for PDP in this cross-sectional study were constrained by the pre-set questions in the surveys. There may have been some potential unobserved confounding factors were not controlled in the generalised linear mixed model. Secondly, the investigation and survey were conducted for managers of public hospitals. Other PDP stakeholders, such as managers of private hospitals and health care authorities were excluded from the study. Thirdly, the relationship between physicians’ support for PDP and that of hospital managers was unclear. More evidence based on quantitative studies and randomized controlled trials may throw light on the unknowns of the mutual interference of stakeholders’ attitudes and behaviors towards PDP. This study used a convenience sampling method which may have resulted in a sample that is not representative of the larger population of public hospital managers in China. In order to yield more evidence based findings, randomized studies in this field are recommended.

## Conclusions

This study found that the majority of public hospital managers in China were not against PDP, rather managers’ support rate for PDP, including support with restrictions, was high. In other words, although public hospital managers in China disagreed with PDP according to various public reports, by implementing PDP with proper conditions left much flexibility and feasibility for the government to achieve optimizing the productivity and capacity of medical resources.

## Supplementary information

**Additional file 1.**

## Data Availability

The dataset and materials used in this study are available from the corresponding author on reasonable request.

## Abbreviations

PDP: Physician Dual Practice
VIE: Valence-Instrumentality-Expectancy
GLMM: Generalized Linear Mixed Model
WHO: World Health Organisation
CI: Confidence Index
OR: Odds Risk
NHS: National Health Service
IT: Information Technology
