# Supplementary material for: Effect of public hospital managers’ risk and gain perception on their attitude towards physician dual practice: a cross-national study in 31 provinces of China
Source: BMC Public Health. 2020 Jul 13;20:1099. doi: 10.1186/s12889-020-09207-1 (PMC7359509; doi:10.1186/s12889-020-09207-1)
Supplement: Supplementary file 1 — Additional file 1. [file 12889_2020_9207_MOESM1_ESM.docx]

**Supplementary material 1**

**Part of Questionnaire for Public Hospital Managers**

**Section A. GENERAL CHARACTERISTICS**

Provinces/autonomous regions/ municipalities:

| Sex | (1) male (2)female |
| --- | --- |
| Age (years) | (1)≦35 (2)36-40 (3)41-45 (4)46-50 (5)51-55 (6)≧56 |
| Major | 1. Medical department (2) Surgical department (3) Pediatrics (4) Gynecology (5) Facial department(6) Dermatological department (7) Plastic surgery department (8) Psychiatry department (9) Chinese medical department (10) General department (11) Others |
| Position | (1) Provincial level(2) City level(3) County level(4) Others |
| Education | (1)PhD(2) Master (3) Bachelor (4) Others |
| Length of service (years) | (1)≦5 (2)6-10 (3)11-15 (4)16-20 (5)21-25 (6)≧26 |
| Institutional category | (1) general hospital(2) special hospital(3) Chinese medicine hospital  (4) Others |
| Location | (1) capital city (2)non-capital city |
| Institutional level | (1) third level (2) second level (3) primary level |
| Institutional territory* | (1) east of China (2) middle of China (3) west of China |
| * East of China including provinces and municipalities: Beijing, Tianjin, Hebei, Liaoning, Shanghai, Jiangsu, Zhejiang, Fujian, Shandong, Guangdong, Hainan; Middle of China including provinces: Shanxi, Jilin, Heilongjiang, Anhui, Jiangxi, Henan, Hubei, Hunan; West of China including provinces and municipalities: Neimenggu, Chognqing, Guangxi, Sichuan, Guizhou, Yunnan, Xizang, Shanxi, Gansu, Qinghai, Ningxia, Xinjiang | |

**Section C Please state your opinion with the following Risks that might raised by Physician Dual Practice for public hospitals.**

1. Physician Dual Practice may bring with more over-provide medical services in public hospital

1. Strongly disagree 2. Disagree 3. Hard to say 4. Agree 5. Strongly agree

1. Physicians involved in Physician Dual Practice may transfer profitable patients from public hospitals to private sectors

1. Strongly disagree 2. Disagree 3. Hard to say 4. Agree 5. Strongly agree

1. Physician Dual Practice may result in predatory behavior and misappropriate use of medical resources

1. Strongly disagree 2. Disagree 3. Hard to say 4. Agree 5. Strongly agree

1. Physician Dual Practice may negatively effect the patients confidence in public hospitals

1. Strongly disagree 2. Disagree 3. Hard to say 4. Agree 5. Strongly agree

1. Physician Dual Practice may raise patients expectation and cause more physician - patient disputes

1. Strongly disagree 2. Disagree 3. Hard to say 4. Agree 5. Strongly agree

1. Physician Dual Practice makes salary administration more difficult

1. Strongly disagree 2. Disagree 3. Hard to say 4. Agree 5. Strongly agree

1. Physician Dual Practice makes it harder to judge duty degree in medical malpractice disputes

1. Strongly disagree 2. Disagree 3. Hard to say 4. Agree 5. Strongly agree

1. Selective referral increases difficulty of medical quality and patient management

1. Strongly disagree 2. Disagree 3. Hard to say 4. Agree 5. Strongly agree

1. Physician Dual Practice will cause time occupation conflict and lead to decline of medical service provision within public hospitals

1. Strongly disagree 2. Disagree 3. Hard to say 4. Agree 5. Strongly agree

1. Physician Dual Practice will negative impact on the development of new technologies and new projects of public hospitals

1. Strongly disagree 2. Disagree 3. Hard to say 4. Agree 5. Strongly agree

1. Physician Dual Practice may make the ownership of scientific research outputs, technological inventions and product patents controversial
2. Strongly disagree 2. Disagree 3. Hard to say 4. Agree 5. Strongly agree

- Are there any other risks that you thought might cause by Physician Dual Practice except for those mentioned above? Please specify.

**Section D Please state your opinion with the following benefits that might brought by Physician Dual Practice for public hospitals.**

1. Physician Dual Practice helps to reduce illegal gains and “under-table” income of physicians in public hospitals

1.Strongly disagree 2. Disagree 3. Hard to say 4. Agree 5. Strongly agree

1. Physician Dual Practice is constructive to promote the salary marketization for physicians in public hospitals

1.Strongly disagree 2. Disagree 3. Hard to say 4. Agree 5. Strongly agree

1. Physician Dual Practice is beneficial in gaining the social recognition and appreciation for the value of physicians

1.Strongly disagree 2. Disagree 3. Hard to say 4. Agree 5. Strongly agree

1. Physician Dual Practice improves health care service access especially for people living in remote and rural areas

1.Strongly disagree 2. Disagree 3. Hard to say 4. Agree 5. Strongly agree

1. Physician Dual Practice raising the overall quality of physicians’ professionalism

1.Strongly disagree 2. Disagree 3. Hard to say 4. Agree 5. Strongly agree

1. Physician Dual Practice urges public hospitals to improve working environment as as to be more competitive

1.Strongly disagree 2. Disagree 3. Hard to say 4. Agree 5. Strongly agree

- Are there any other gains that you thought might brought by Physician Dual Practice except for those mentioned above? Please specify.

**Section E Please state your attitude towards physician dual practice in public hospitals.**

1. For the physician dual practice in public hospitals, you think (single choice)

(1) Physician Dual Practice should be totally prohibited

(2) Physician Dual Practice should be allowed with restrictions

(3) Physician Dual Practice should be totally allowed
